# High-Resolution Spatiotemporal-Coded Differential Eddy-Current Array Probe for Defect Detection in Metal Substrates

**DOI:** 10.3390/s26020537

**Published:** 2026-01-13

**Authors:** Qi Ouyang, Yuke Meng, Lun Huang, Yun Li

**Affiliations:** 1 School of Automation, Chongqing University, Chongqing 400044, China; 202413131054t@stu.cqu.edu.cn (Y.M.);; 2 Southwest Institute of Technology and Engineering, China South Industries Group, Chongqing 400039, China

**Keywords:** differential eddy-current array probe, high-resolution imaging, spatiotemporal coding, defect detection, quantitative evaluation

## Abstract

To address the problems of weak geometric features, low signal response amplitude, and insufficient spatial resolvability of near-surface defects in metal substrates, a high-resolution spatiotemporal-coded eddy-current array probe is proposed. The probe adopts an array topology with time-multiplexed excitation and adjacent differential reception, achieving a balance between high common-mode rejection ratio and high-density spatial sampling. First, a theoretical electromagnetic coupling model between the probe and the metal substrate is established, and finite-element simulations are conducted to investigate the evolution of the skin effect, eddy-current density distribution, and differential impedance response over an excitation frequency range of 1–10 MHz. Subsequently, a 64-channel M-DECA probe and an experimental testing platform are developed, and frequency-sweeping experiments are carried out under different excitation conditions. Experimental results indicate that, under a 50 kHz excitation frequency, the array eddy-current response achieves an optimal trade-off between signal amplitude and spatial geometric consistency. Furthermore, based on the pixel-to-physical coordinate mapping relationship, the lateral equivalent diameters of near-surface defects with different characteristic scales are quantitatively characterized, with relative errors of 6.35%, 4.29%, 3.98%, 3.50%, and 5.80%, respectively. Regression-based quantitative analysis reveals a power-law relationship between defect area and the amplitude of the differential eddy-current array response, with a coefficient of determination $R^{2}=0.9034$ for the bipolar peak-to-peak feature. The proposed M-DECA probe enables high-resolution imaging and quantitative characterization of near-surface defects in metal substrates, providing an effective solution for electromagnetic detection of near-surface, low-contrast defects.

## 1. Introduction

In aerospace equipment, aluminum alloy components are widely used as key structural materials for skins, connectors, and load-bearing frames. Under cyclic service loads and coupled environmental effects, the mechanical performance of the metal substrate may degrade, leading to the initiation of imperceptible localized defects beneath the surface [1,2]. Owing to their weak geometric features and low signal response amplitudes, high-resolution detection and quantitative characterization of near-surface defects in metal substrates have become one of the critical challenges that urgently need to be addressed in the field of structural health monitoring (SHM) [3,4].

In response to the nondestructive testing (NDT) requirements for surface defects in metal substrates, extensive research efforts have been carried out worldwide. Currently, commonly used NDT techniques mainly include ultrasonic testing [5], radiographic testing [6], infrared thermography [7], and electromagnetic testing [8]. Among these methods, electromagnetic nondestructive testing is regarded as a promising approach for addressing near-surface defect detection in metal substrates, owing to its inherently high sensitivity to defects in conductive materials near the surface [9,10,11]. Zhang Na, Ye Chaofeng, and their research teams [12,13,14,15] proposed a three-phase excited eddy-current probe incorporating a high-resolution tunnel magnetoresistance (TMR) sensor array and spatially interleaved circular coil configurations. By fabricating an array prototype probe using flexible PCB technology, rapid scanning detection without multiplexing circuits was achieved, significantly improving the signal-to-noise ratio and localization imaging accuracy for micro-defects. Huang et al. [16] developed an improved multi-frequency eddy-current array sensor, enabling the extraction of defect responses with different characteristic scales through multi-frequency excitation strategies. She et al. [17] further enhanced defect depth evaluation accuracy by combining a four-coil excitation array with an improved data-processing model. Ma et al. [18] effectively suppressed lift-off effects using a synergistic signal-conditioning method based on bridge and transformer coupling, while Zhao et al. [19] improved the resolution of eddy-current displacement sensors to the sub-nanometer scale by suppressing excitation voltage noise. Su et al. [20] proposed a near- and remote-field pulsed eddy-current integrated testing method (NRPEC) for detecting buried defects in multilayer conductors and analyzed the electromagnetic response characteristics of hidden defects using a symmetric probe configuration.

In recent years, excitation schemes in eddy-current testing have evolved toward spatiotemporal-coded sampling. By introducing coded excitations such as swept-frequency signals, chirp signals, or pseudorandom sequences, combined with signal-processing strategies such as pulse compression, the effective injected energy can be enhanced without sacrificing spatial resolution, thereby improving the signal-to-noise ratio and defect discriminability [21]. Meanwhile, eddy-current array (ECA) data inherently exhibit both spatial and temporal dimensions. Existing studies have introduced mechanisms such as spatiotemporal attention to jointly model temporal–spatial array data, effectively enhancing the recognition capability for multi-scale defects [22]. In addition, swept-frequency electromagnetic excitation and spatiotemporal feature extraction methods have been applied to electromagnetic–thermal coupled imaging and tomographic characterization, providing new approaches for the quantitative analysis of defects in complex structures [23].

With respect to quantitative defect characterization, research efforts have increasingly focused on geometric parameter inversion and accuracy evaluation. Previous studies have demonstrated that quantitative reconstruction of defect geometric parameters can be directly achieved from multi-channel responses, enabling quantitative outputs for imaging-based inspections [24]. For localized structural defects, pulsed eddy-current testing (PECT) combined with feature extraction and inversion algorithms has been shown to achieve quantitative evaluation of defect parameters, verifying the stability and feasibility of the method [25]. Moreover, traditional statistical approaches establish mapping relationships between impedance signals and defect dimensions through linear regression and related techniques, exhibiting good quantitative detection performance in experiments. Regression models based on deep learning have also been introduced into eddy-current signal inversion tasks, achieving high prediction accuracy on multi-defect datasets; for example, residual convolutional networks have reported defect depth classification accuracies exceeding 93% [26].

In summary, existing methods still face challenges in simultaneously balancing detection sensitivity, imaging resolution, and noise suppression [27,28]. To address these issues, this paper proposes a novel multiplexed differential eddy-current array probe with spatiotemporal coding features and an equilateral triangular configuration (Multiplexed Differential Eddy Current Array Probe with Equilateral Triangular Configuration, M-DECA Probe). By introducing an equilateral triangular array topology to optimize electromagnetic coupling and combining a time-multiplexed excitation–reception mechanism, the proposed probe achieves an effective balance between high common-mode rejection capability inherent to differential structures and high-density spatial sampling with manageable system complexity. Experimental results verify its effectiveness in imaging and quantitative characterization of near-surface defects in metal substrates. Based on pixel-to-physical dimension mapping, quantitative evaluation of the lateral dimensions of near-surface defects is conducted, providing experimental evidence for the quantitative performance assessment of differential eddy-current array imaging. This work offers a novel field-modulation excitation strategy for high-resolution imaging of near-surface, low-contrast defects.

## 2. Study of the Detection Mechanism of Eddy-Current Array Probes

### 2.1. Structure of the M-DECA Probe

**Figure 1:**
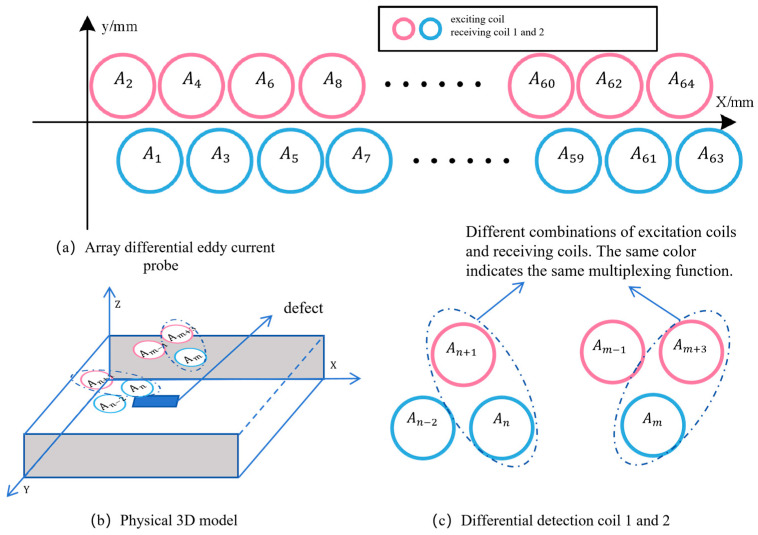
Schematic diagram of the multiplexed differential eddy-current array probe (M-DECA) with an equilateral triangular configuration.

Based on the differences in magnetic permeability associated with surface defects in metal substrates, variations in the internal magnetic flux are induced, leading to changes in the electromagnetic field and the generation of electromagnetic induction voltages near the surface of the metal substrate. On this basis, a novel multiplexed differential eddy-current array probe with spatiotemporal coding features and an equilateral triangular configuration (M-DECA Probe) is designed. The probe adopts a sensing-unit topology with high spatial sampling density and consists of 64 channels of symmetrically arranged excitation/detection coils in an equilateral triangular layout. The structure of the probe is illustrated in Figure 1a, showing the time-multiplexed excitation and adjacent differential reception architecture of the M-DECA probe. Figure 1b,c depict the dynamic operating sequence of the probe at two different working instants, illustrating the electrical connections and activation states. Here, $A_{m}$ and $A_{n}$ denote generic coil position identifiers, indicating that the excitation position of the probe shifts with time during scanning.

The probe operates in a time-multiplexed excitation and adjacent multiplexed reception mode, in which high-frequency sinusoidal excitation signals are sequentially applied to coils 1–64, while two adjacent coils are multiplexed as receiving units to realize differential excitation–reception coupling. This configuration enables real-time acquisition of eddy-current field perturbation signals induced by defects. By successively switching the excitation position, the entire array is spatially equivalent to a series of overlapping differential sensing units, which strengthens the complementary electromagnetic coupling between the excitation field and the induced field. As a result, the proposed design enhances common-mode noise suppression under complex industrial environments and improves the uniformity of the probe’s electromagnetic field sensitivity in the planar direction.

### 2.2. Equivalent Circuit Model of the M-DECA Probe

**Figure 2:**
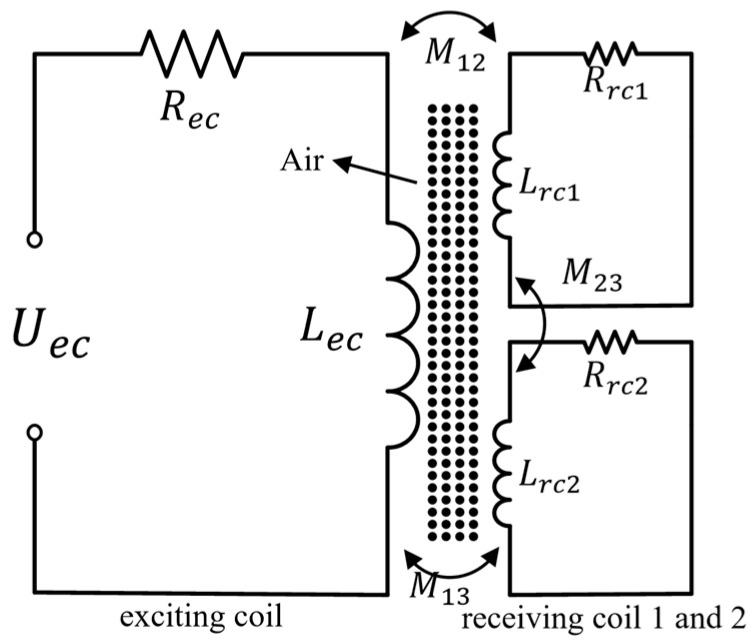
Equivalent circuit of the M-DECA differential eddy current probe.

Differential eddy current sensors exhibit higher resolution, enhanced sensitivity, and improved robustness compared with conventional absolute eddy current sensors [29,30]. To investigate the dynamic electromagnetic coupling between the excitation coil and the heterogeneous structure of the metallic substrate, and to elucidate the generation mechanism of differential signal characteristics and the corresponding electromagnetic topological response, an equivalent circuit model of the M-DECA probe based on a three-winding transformer is developed, as illustrated in Figure 2.

Figure 2 illustrates the equivalent circuit model of the M-DECA probe. The excitation coil is modeled as the primary winding of a transformer, while the two receiving coils are represented as secondary windings 1 and 2. The primary magnetic flux path encompasses the test object and the air medium, collectively equivalent to the transformer’s core.

Anomalous internal structures within the metal substrate and variations in its permeability induce abrupt changes in the local magnetic reluctance of the circuit and the impedance of the secondary eddy-current loop. This disrupts the electromagnetic symmetry of the original excitation-reception path, leading to alterations in the system’s impedance matrix and the induced electromagnetic voltage. Consequently, a high-sensitivity differential voltage is output by the differential probe. Based on Faraday’s law of electromagnetic induction and Kirchhoff’s circuit laws, a mathematical model for the multi-winding electromagnetic coupling system and the equivalent equations for the single-excitation dual-reception differential probe are established. This model reveals the physical essence of electromagnetic energy redistribution under defect-induced perturbations and provides the theoretical and technical foundation for achieving high signal-to-noise ratio and high-resolution imaging detection of near-surface defects in metal substrates.

The mathematical model for the multi-winding electromagnetic coupling is formulated as follows:

(1) $$\left[\begin{array}{c} U_{\mathrm{ec}}\\ U_{\mathrm{rc}1}\\ U_{\mathrm{rc}2}\\ 0 \end{array}\right]=\left[\begin{array}{cccc} Z_{1} & j\omega M_{12} & j\omega M_{13} & j\omega M_{1g}\\ j\omega M_{21} & Z_{2} & j\omega M_{23} & j\omega M_{2g}\\ j\omega M_{31} & j\omega M_{32} & Z_{3} & j\omega M_{3g}\\ j\omega M_{g1} & j\omega M_{g2} & j\omega M_{g3} & Z_{g} \end{array}\right]\left[\begin{array}{c} i_{1}\\ i_{2}\\ i_{3}\\ i_{g} \end{array}\right]$$

where $U_{\mathrm{ec}}$ is the excitation voltage source, Asin(wt); $U_{\mathrm{rc}1}$, ${\;U}_{\mathrm{rc}2}$ are the induced voltages in the receiving coils; $I_{i}(i=1,2,3,g)$ denotes the currents in loops 1, 2, 3, and the eddy current loop g, respectively. Notably, currents ${\dot{I}}_{2}$, ${\dot{I}}_{3}$ are considered open-circuit currents; ${\dot{I}}_{g}$ is the induced eddy current; $M_{11},M_{12},M_{23}$ represent the mutual inductances between the excitation and receiving coils (1–2, 1–3) and between the two receiving coils (2–3), respectively; $M_{ig}$ (i=1,2,3) is the mutual inductance between coil i and the eddy current loop g; $Z_{k}$ is the self-impedance of each winding, defined as $Z_{k}=R_{k}+j\omega L_{k}$.

Given the high input impedance of the receiving coils, which can be treated as open circuits, the currents satisfy ${\dot{I}}_{2}={\dot{I}}_{3}\approx 0$. Under this condition, Equation (1) can be simplified as

(2) $$\begin{array}{l} \begin{array}{c} U_{\mathrm{rc}1}=j\omega M_{21}i_{1}+j\omega M_{2g}i_{g} \end{array}\\ U_{\mathrm{rc}2}=j\omega M_{31}i_{1}+j\omega M_{3g}i_{g} \end{array}$$

The relationship between $i_{1}$ and $i_{g}$, derived from the loop g Equation (1), is

(3) $$0=j\omega M_{g1}i_{1}+j\omega M_{g2}i_{2}+j\omega M_{g3}i_{3}+Z_{g}i_{g}$$

For the case of ${\dot{I}}_{2}={\dot{I}}_{3}\approx 0$, the relation between $i_{g}$ and $i_{1}$ is

(4) $${\dot{I}}_{g}=-\frac{j\omega M_{g1}}{Z_{g}}{\dot{I}}_{1}$$

By substituting Equation (4) into Equation (2), we obtain the induced voltages $U_{rc1}$ and $U_{rc2}$:

(5) $$\begin{array}{l} U_{\mathrm{rc}1}=j\omega M_{21}i_{1}-\omega^{2}\frac{M_{2g}M_{g1}}{Z_{g}}i_{1},\\ U_{\mathrm{rc}2}=j\omega M_{31}i_{1}-\omega^{2}\frac{M_{3g}M_{g1}}{Z_{g}}i_{1} \end{array}$$

Thus, the differential voltage is given by

(6) $$\begin{array}{l} U_{\mathrm{rc}}=U_{\mathrm{rc}1}-U_{\mathrm{rc}2}\\ =j\omega (M_{21}-M_{31})i_{1}-\omega^{2}\frac{(M_{2g}-M_{3g})M_{g1}}{Z_{g}}i_{1} \end{array}$$

The impedance of the differential coil is derived by solving for current $i_{1}$ in Equation (6):

(7) $$Z_{d}=j\omega (M_{21}-M_{31})-\omega^{2}\frac{(M_{2g}-M_{3g})M_{g1}}{Z_{g}}$$

The real part, $Re(Z_{d})$, and the imaginary part, Im(Zd), of the impedance are given by

(8) Re(Zd)=−ω2(M2g−M3g)Mg1RgRg2+Xg2,Im(Zd)=ω(M21−M31)+ω2(M2g−M3g)Mg1XgRg2+Xg2

In Equation (8), the real part corresponds to the resistance component, which quantifies the energy loss of the eddy current and is governed by the electrical conductivity σ of the metal. The imaginary part represents the reactance component, characterizing the energy storage within the eddy current’s magnetic field, which is influenced by the geometry of the eddy current path.

The presence of anomalous structures within the metal substrate alters key parameters in the transformer model, including the mutual inductances $M_{1g}$, $M_{2g}$, $M_{3g}$ and the eddy-current loop parameters $R_{g}$, $L_{g}$. This perturbation disrupts the system’s electromagnetic balance, causing the differential probe to output an imbalance signal, $U_{rc}$. The differential coil impedance, $Z_{d}$, is subsequently derived from $U_{rc}$. Ultimately, the geometry of the anomalous structure is inverted by analyzing this impedance signal.

### 2.3. Finite Element Analysis of the Probe Model

#### 2.3.1. Eddy Current Detection Principle

The mathematical model for simulating the equilateral triangular differential array probe is established based on Maxwell’s equations. The time-varying magnetic field generates a vortex electric field, expressed as

(9) $$\nabla \times E=-\frac{\partial B}{\partial t}$$

Ampere’s Loop Law:

(10) $$\nabla \times H=J+\frac{\partial D}{\partial t}$$

Gauss’s law of electricity:

(11) ∇⋅D=ρ

Gauss’s Law of Magnetism:

(12) ∇⋅B=0

The principal constitutive equations:

(13) B=μH,    J=σE,    D=ϵE

In Equation (13), μ, σ, ϵ denote the magnetic permeability, electrical conductivity, and dielectric constant of the material, respectively.

Given the probe’s operating frequency range and the high conductivity of the test object, the conduction current density J dominates the displacement current density $\frac{\partial D}{\partial t}$, satisfying σ/(ωϵ)≫1. Consequently, the displacement current term can be neglected.

Consistent with Gauss’s law for magnetism, ∇⋅B = 0, the magnetic flux density is defined as the curl of the magnetic vector potential, B = ∇ × A. The electric scalar potential ϕ is also introduced.

Substituting this definition into Equation (9), which describes the electric field induced by the time-varying magnetic field, yields:

(14) $$\nabla \times E=-\frac{\partial }{\partial t}(\nabla \times A)=-\nabla \times \left(\frac{\partial A}{\partial t}\right)$$

It follows that: $\nabla \;\times \;\left(E\;+\;\frac{\partial A}{\partial t}\right)=0$.

Consequently, the electric field strength can be expressed as

(15) $$E=-\nabla \phi -\frac{\partial A}{\partial t}$$

Combining the constitutive relation J = σE with Ampère’s law ∇ × H = J and the relation B = μH, we obtain

(16) $$\nabla \times \left(\frac{1}{\mu }\nabla \times A\right)=J$$

The current density J comprises the source current $J_{s}$ in the probe’s excitation coil and the eddy current $J_{e}$ induced in the conductive medium:

(17) $$J\;=\;J_{s}\;+\;J_{e}\;=\;J_{s}\;+\;\sigma E$$

Substituting the expression for the electric field strength from Equation (15) yields

(18) $$J=J_{s}+\sigma \left(-\nabla \phi -\frac{\partial A}{\partial t}\right)$$

Substituting Equation (18) into Equation (16) yields the coupled equations for the magnetic vector potential A and the electric scalar potential ϕ:

(19) $$\nabla \times \left(\frac{1}{\mu }\nabla \times A\right)+\sigma \frac{\partial A}{\partial t}+\sigma \nabla \phi =J_{s}$$

In a homogeneous, linear, and isotropic medium with no free charge region (ρ = 0), the electric scalar potential ϕ satisfies Laplace’s equation $\nabla^{2}\phi \;=\;0$. Assuming a constant solution for ϕ, the governing equation for the eddy-current field in the  A-ϕ formulation is derived:

(20) $$\nabla \times \left(\frac{1}{\mu }\nabla \times A\right)+\sigma \frac{\partial A}{\partial t}=J_{s}$$

For sinusoidal excitation, Equation (20) can be expressed in complex form:

(21) $$\nabla \times \left(\frac{1}{\mu }\nabla \times A\right)+j\omega \sigma A=J_{s}$$

In Equation (21), the term $\nabla \;\times \;\left(\frac{1}{\mu }\nabla \;\times \;A\right)$ represents the storage of magnetic energy; the term jωσA quantifies the eddy-current loss in the conductor, which is proportional to the conductivity σ and the angular frequency ω; and $J_{s}$ is the source current density of the probe excitation.

#### 2.3.2. Simulation Parameter Setting

**Figure 3:**
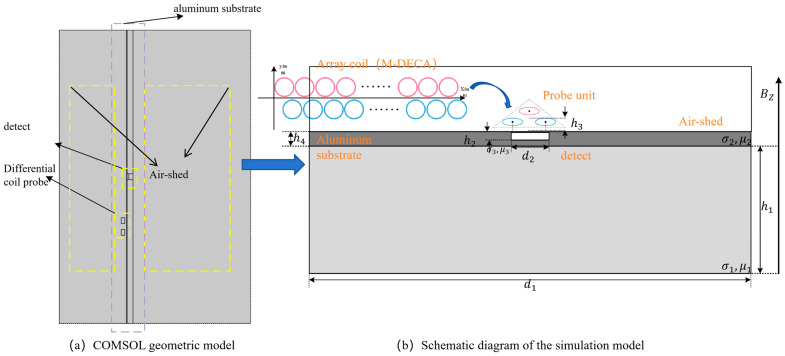
Finite element model of the M-DECA probe: (**a**) COMSOL geometric model; (**b**) Schematic diagram of the simulation model.

To validate the equivalent circuit model of the differential probe with single-excitation dual-reception, a two-dimensional (2D) axisymmetric numerical model was established using COMSOL Multiphysics software 6.2, as illustrated in Figure 3. The multiphysics simulation utilized the Magnetic Fields (mf) interface within the AC/DC Module, with the analysis type set to Frequency Domain. The specific parameters of the simulation model are listed in Table 1, It should be noted that the conductivity values assigned to the air domain and the defect domain in the model are equivalent parameters adopted for numerical modeling, rather than the actual physical properties of any specific material.

#### 2.3.3. Analysis of Simulation Experiment Results

The defect depth on the metal substrate surface was fixed at 100 μm. The probe was positioned at five representative locations, including both sides of the defect, the inner boundaries, and the defect center. Excitation frequencies of 1 kHz, 10 kHz, 100 kHz, 1 MHz, and 10 MHz were employed with a decade frequency interval. The lift-off distance between the probe and the specimen surface was maintained at 1 mm.

**Figure 4:**
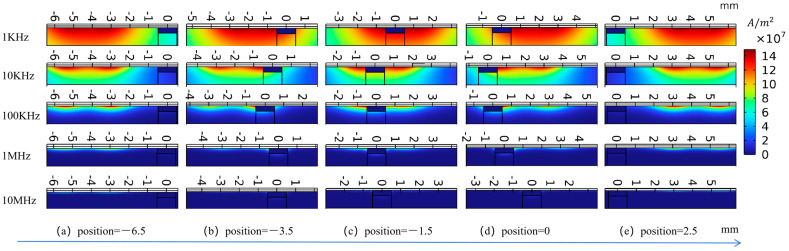
Distribution of eddy current density at different excitation frequencies.

Figure 4 illustrates the distribution of eddy-current density at different excitation frequencies. It can be observed that the figure illustrates a three-dimensional relationship among excitation frequency, spatial position, and eddy-current density, indicating that the eddy-current density distribution is highly sensitive to both the excitation frequency and the spatial position of the probe. As the excitation frequency increases, the amplitude of the eddy-current density is significantly enhanced. Under low-frequency excitation, the eddy current exhibits a relatively large longitudinal penetration depth, enabling effective penetration into the metal substrate; however, the local perturbations induced by defects are relatively weak, resulting in a smoother eddy-current distribution and limited spatial resolution. At an excitation frequency of 1 kHz, the eddy-current density is on the order of 10^7^ A/m^2^, whereas it increases to approximately 10^10^ A/m^2^ at 10 MHz, demonstrating that high-frequency excitation can significantly strengthen the electromagnetic response within the metal substrate.

As the probe moves along the scanning path from −6.5 mm to 2.0 mm, the mutual interference of eddy-current density between different spatial positions gradually decreases. When the excitation frequency exceeds 10 kHz, pronounced defect-induced eddy-current density discontinuities become evident, with local variations reaching the order of 10^11^ A/m^2^. These results indicate that the differential probe exhibits high electromagnetic response sensitivity and strong spatial discriminability for near-surface defects. Moreover, intermediate-frequency excitation conditions achieve a favorable balance between penetration capability into the metal substrate and sensitivity to near-surface defects, providing a theoretical basis for excitation frequency selection in subsequent M-DECA probe imaging.

**Figure 5:**
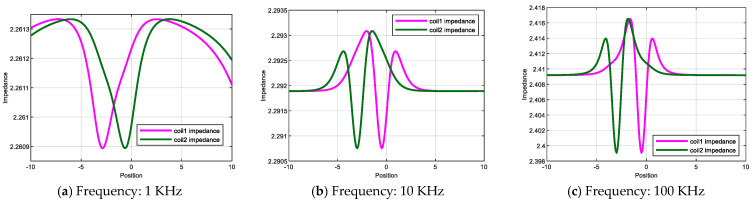

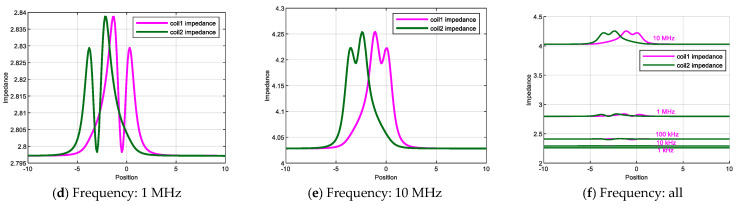
Complex impedance response characteristics at different excitation frequencies. (**a**–**e**) Impedance characteristics at five excitation frequencies; (**f**) comparative impedance across all frequencies.

Figure 5 shows the complex impedance response characteristics of the differential coils under different excitation frequencies. At excitation frequencies of 1 kHz, 10 kHz, 100 kHz, 1 MHz, and 10 MHz, the corresponding baseline impedance values are approximately 2.261 Ω, 2.292 Ω, 2.409 Ω, 2.80 Ω, and 4.03 Ω, respectively. As the excitation frequency increases, the impedance baseline rises accordingly, and the amplitude of defect-induced impedance perturbations is also enhanced. At low frequencies, only weak variations on the order of $10^{-4}–10^{-3}\;$ Ω are observed, whereas at MHz-level excitation frequencies, the perturbation magnitude increases to the order of $10^{-1}–10^{0}$ Ω. Consequently, defect-related peak–valley structures become clearly distinguishable under intermediate- and high-frequency excitation conditions.

In addition, a stable spatial offset between the peak and valley positions is observed along the scanning axis, indicating asymmetric electromagnetic perturbations induced by defects on adjacent coils. This behavior contributes to enhanced detection sensitivity and improved spatial discriminability of the probe for near-surface defects.

## 3. Experimental Verification

### 3.1. Experimental Test System

#### 3.1.1. Excitation and Receiving Circuit of the System

To improve the scanning efficiency of the eddy-current array probe under time-multiplexed excitation, a low-phase-noise, programmable excitation and differential receiving front-end circuit is designed in this study. The excitation signal is generated by a direct digital synthesizer (DDS, AD9834, Analog Devices, Wilmington, MA, USA), which enables multi-frequency sinusoidal signal output. In the fixed-frequency mode, the DDS continuously provides a stable excitation signal, and its transient response is mainly limited by the subsequent analog circuitry. The excitation signal undergoes current-to-voltage conversion through a resistor and, together with a parallel capacitor, forms a low-pass filtering network to suppress high-frequency spikes and harmonic components, thereby producing a sinusoidal excitation signal with stable amplitude and high spectral purity.

**Figure 6:**
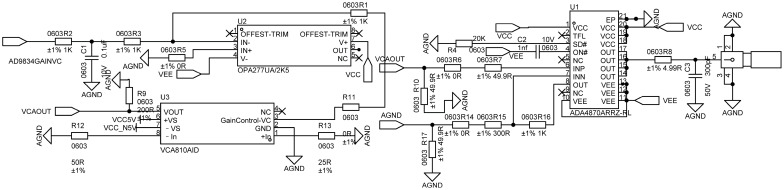
Schematic diagram of the excitation circuit.

The amplified excitation signal is sequentially applied to individual excitation coils in the M-DECA probe via a multi-channel switching network, ensuring that only one excitation channel is active at any given time. This configuration guarantees the determinacy and repeatability of the excitation magnetic-field topology. Moreover, it effectively avoids electromagnetic coupling and synchronization interference that may arise under multi-channel parallel excitation. The circuit design of the excitation unit is illustrated in Figure 6.

**Figure 7:**
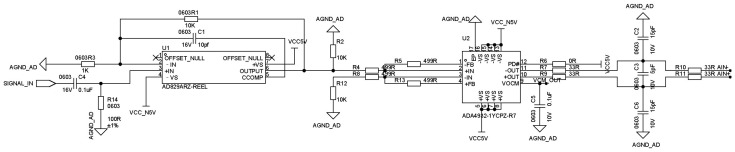
Schematic diagram of the receiving circuit.

Figure 7 illustrates the circuit architecture of the receiving unit. The system employs high-speed analog components to condition the induced signals from the coils, including wideband amplifiers and differential ADC drivers, whose settling times are on the nanosecond scale and are therefore much shorter than the overall detection cycle of the system. The conditioned differential signals are sampled by a high-speed analog-to-digital converter AD9265. In continuous operation mode, a sampling window on the order of microseconds is sufficient to complete amplitude extraction.

The sampling period for each individual channel is set to 1 ms, and a complete scan of all 64 channels of the M-DECA probe requires approximately 64 ms, achieving a balance between detection efficiency and spatial resolution. The detailed circuit parameters are listed in Table 2.

The M-DECA probe implements electronic channel switching based on a multi-channel switch, with a channel switching time of approximately 2 ns, which is much shorter than the time required for mechanical scanning of the probe. This architecture reduces the circuit complexity of the array probe while providing a stable and controllable signal foundation for subsequent differential modeling and imaging inversion of array signals.

#### 3.1.2. Experimental Set Up

**Figure 8:**
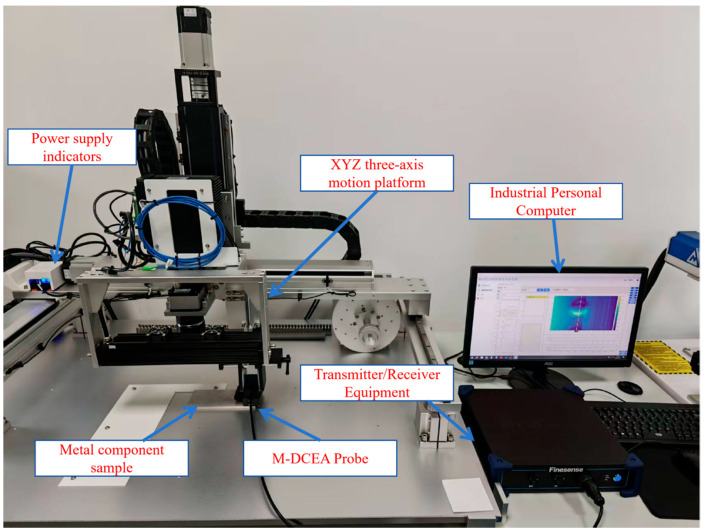
Experimental platform for transient eddy current testing using the M-DECA probe.

To validate the simulation results and verify the feasibility of the proposed M-DECA array probe, an experimental eddy current detection system was established, as shown in Figure 8. The experimental platform comprises an industrial control computer, an excitation/receiving unit, an XYZ three-axis motion stage, the fabricated M-DECA probe, and an aluminum alloy specimen containing predefined defects.

**Figure 9:**
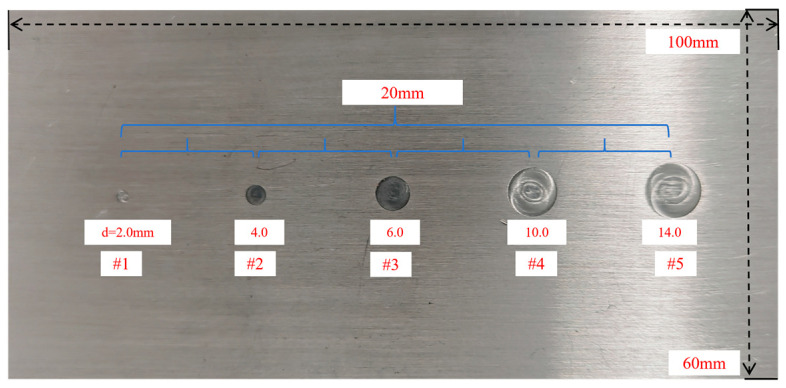
Multiscale morphology of anomalous structures on the aluminum alloy substrate.

Based on the simulation results, frequency-sweeping experiments were conducted with excitation frequencies ranging from 10 kHz to 1 MHz, and the excitation amplitude was set to 12 V. The test specimen measures 100 mm × 60 mm × 5 mm and is made of aluminum alloy. Artificial circular defects with diameters of 2.0 mm, 4.0 mm, 6.0 mm, 10.0 mm, and 14.0 mm (Defects #1–#5) were machined on the surface, arranged in increasing order with a 20 mm center-to-center spacing, All defects had a uniform depth of 100 μm. The dimensional layout of the defected specimen is illustrated in Figure 9.

**Figure 10:**
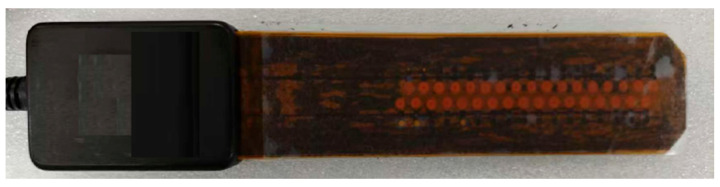
M-DECA probe.

The structural configuration of the M-DECA probe is illustrated in Figure 10. The array-type differential probe adopts a flexible printed circuit board (FPCB) design, in which the excitation and receiving coils are embedded with an arrangement identical to that shown in Figure 1. The lift-off distance between the probe and the specimen surface is maintained at 1 mm to ensure stable electromagnetic coupling and consistent detection sensitivity.

### 3.2. Experimental Results and Discussion

A self-developed 64-channel M-DECA probe was employed to perform spatiotemporally coded excitation and response signal acquisition on the test specimen. The responses of different channels exhibit nonuniform characteristics in the spatiotemporal sequence. In the initial stage $X_{1}$, the channels show low-amplitude fluctuations with an average voltage of $U_{av}<0.002\;V$, during which the system performs spatial calibration of the array channels and establishes a stable operating state. As the scanning progresses to $X_{t}$, anomalous structures within the metal substrate are detected by the probe, and the signal amplitudes of all channels increase significantly. The alteration of the magnetic flux paths caused by internal structural anomalies leads to output imbalance in the differential probe.

**Figure 11:**
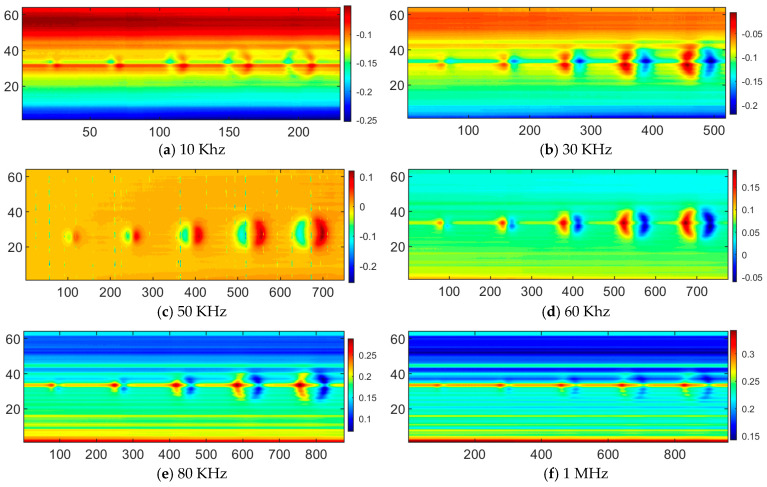
Array eddy-current responses obtained under different excitation frequencies.

Figure 11 presents the eddy-current responses obtained under different excitation frequencies. In the low-frequency range, defect responses are relatively weak but exhibit clear spatial localization characteristics. With increasing excitation frequency, the response amplitude is markedly enhanced, and a pronounced bipolar pattern emerges, indicating that the eddy-current perturbations become increasingly influenced by defect boundaries. In the intermediate-frequency range, the defect responses exhibit high contrast and spatial features that are geometrically consistent with the defect layout, achieving an optimal balance between sensitivity and geometric fidelity. At high frequencies, although the peak amplitude of the defect response continues to increase, the spatial distribution of the response becomes progressively broader and more blurred. This results in geometric distortion and reduced discriminability among defects of different sizes, indicating that excessively high excitation frequencies can degrade the reliability of defect imaging and quantitative characterization.

In addition, defect contour information is extracted to determine the coordinates of the two endpoints of the equivalent circular defect boundary along the scanning direction. By combining the pixel-to-physical dimension conversion relationship given in Equation (23), the optimal equivalent diameters of defects at all excitation frequencies are further calculated. Subsequently, a quantitative evaluation of the deviation between the inverted defect sizes and the actual dimensions is performed, and the corresponding error results are summarized in Table 3.

The physical dimension corresponding to a single pixel along the scanning direction can be expressed as

(22) $$S=v\times {(t}_{1}+\left(t_{2}+\frac{N}{f}\right)\times n)$$

where S denotes the physical distance between adjacent pixels along the scanning direction (mm); v is the scanning speed (mm/s), which is set to 1 mm/s in the experiments; $t_{1}$ represents the scanning cycle interval (s), set to 0.001 s; $t_{2}$ is the timing delay (s), also set to 0.001 s; N denotes the number of sampling cycles, set to N = 60; f is the frequency (Hz); and n is the number of sensor elements, set to n = 64.

(23) $$\varepsilon =|\frac{D_{true}-D_{inv}}{D_{true}}|\times 100\%$$

where $D_{true}$ denotes the actual diameter of the defect, $D_{inv}$ represents the inverted estimated defect diameter, and ε is the relative error of the defect size.

The results in Table 3 indicate that, under an excitation frequency of 50 kHz, the relative error between the calculated equivalent defect diameters and the actual specimen dimensions is minimized. The relative errors for defects #1 to #5 are 6.35%, 4.29%, 3.98%, 3.50%, and 5.80%, respectively. These results demonstrate that the defect size inversion accuracy at this excitation frequency is relatively high and satisfies the detection requirements. In addition, the experimental results confirm that the M-DECA probe exhibits high spatial resolution and high sensitivity in defect detection, which is consistent with the previously obtained numerical simulation results.

**Figure 12:**
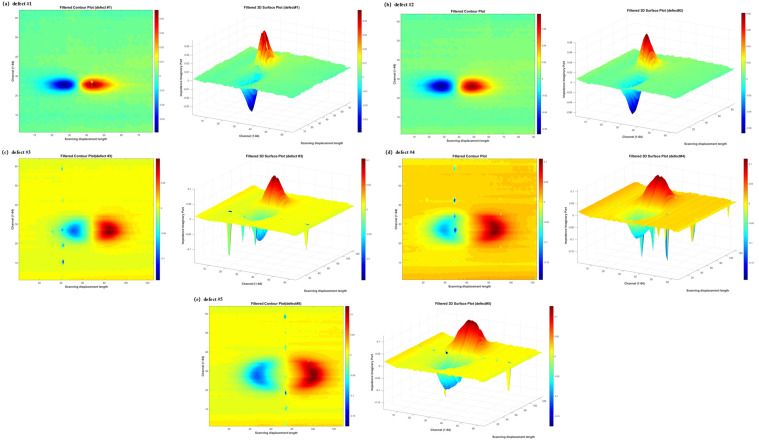
Visualization of filtered defect response images for defects #1–#5.

**Figure 13:**
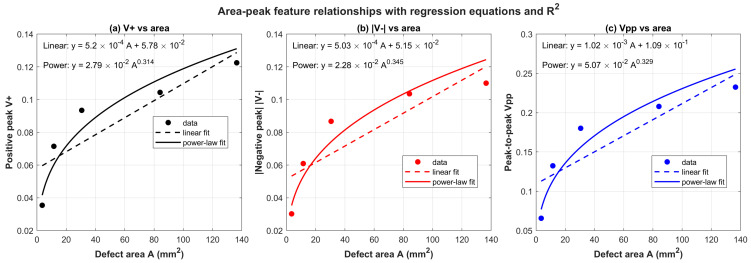
Relationship between the peak voltage and the area.

Figure 12 shows the isolated responses of defects #1–#5, reflecting the spatial mapping relationship between the three-dimensional response domain and the actual physical defect space. On this basis, the differences in amplitude distribution and spatial extent among the isolated responses of different defects provide a direct foundation for establishing a quantitative relationship between the detection response characteristics and the defect geometric dimensions as well as the equivalent defect area. Therefore, a regression fitting algorithm is designed to quantitatively evaluate the correlation between the detection response features and the defect area, with the corresponding results shown in Figure 13. Under the 50 kHz excitation condition, the defect area exhibits a significant monotonic correlation with the peak characteristics of the array eddy-current response. The positive peak is denoted as $V_{+}$, the magnitude of the negative peak is denoted as $\left|V_{-}\right|$, and the bipolar composite feature (peak-to-peak value) is defined as $V_{pp}$ = $V_{+}$ + $\left|V_{-}\right|$.

The unipolar feature $V_{+}$ exhibits a strong positive correlation with the defect area, with a coefficient of determination of $R^{2}\approx 0.9022$, while the correlation between $\left|V_{-}\right|$ and the defect area yields $R^{2}\approx 0.8951$. The bipolar composite indicator $V_{pp}$ shows the strongest correlation with the defect area, with $R^{2}\approx 0.9034$. These results indicate that, under bipolar response conditions, a single-sided peak characterizes only localized extreme perturbations and is therefore susceptible to local field concentration, noise, and non-uniformity of the sensitive region. In contrast, the peak-to-peak value $V_{pp}$ integrates information from both the positive and negative lobes, providing a more comprehensive representation of the overall eddy-current perturbation induced by the defect. As a result, $V_{pp}$ offers more stable characterization of defect area and exhibits stronger correlation.

In addition, the peak signal of the probe demonstrates a typical behavior of “sensitive growth followed by gradual saturation.” In the small-area range of $A\approx 3.55\;to\;30.56\;{\mathrm{mm}}^{2}$, the peak amplitudes increase rapidly, with V+ rising from 0.0354 to 0.0934, $\left|V_{-}\right|$ increasing from 0.0302 to 0.0867, and $V_{pp}$ increasing from 0.0657 to 0.1802, indicating that defect expansion effectively enhances eddy-current path perturbations. In contrast, in the larger-area range of $A\approx 84.11\;to\;136.52\;{\mathrm{mm}}^{2}$, the growth of the peak amplitudes becomes noticeably slower.

**Figure 14:**
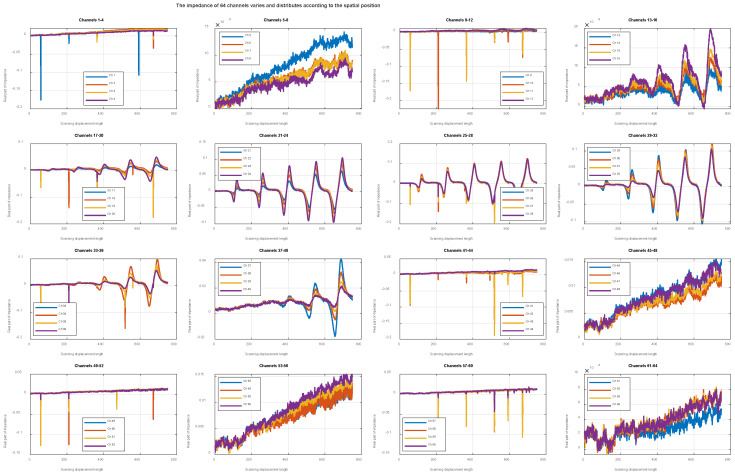
Distribution of impedance responses across the 64 array channels.

The spatial configuration and topological structure of the probe play a decisive role in the acquisition of array eddy-current signals. Within the imaging space constructed based on spatial coding, the temporal responses of channels 1–64 are unfolded on a channel-by-channel basis, revealing significant differences in defect response sensitivity among different channels. Figure 14 illustrates the distribution of impedance responses across the 64 array channels. As shown in Figure 14, the channel responses exhibit pronounced spatial non-uniformity; defect responses appear as locally concentrated features in the channel domain rather than being uniformly distributed across all channels. Moreover, each channel of the probe exhibits stable and repeatable bipolar defect response patterns. The overall array response demonstrates a clear spatial topological correlation with the scanning displacement and maintains geometric consistency with the actual defect distribution.

Specifically, channels 1–4, 9–12, 41–44, and 49–52 exhibit relatively low response amplitudes, whereas channels 21–28, 29–36, and 37–40 show clear and repetitive impedance peak–valley structures. The peaks and valleys appear in paired form along the scanning direction, and the responses of different channels to the same defect remain stably aligned along the scanning axis. These results indicate that the M-DECA probe possesses intrinsic spatial coding and topological mapping capabilities.

Channel 28 in the M-DECA probe exhibits a pronounced defect response. By performing spatial-domain unfolding analysis of the voltage acquisition signal from this channel, a distinct impedance peak can be observed at x = 120. After physical coordinate calibration, this peak position corresponds precisely to the actual spatial location on the right side of the center of defect #1. Under the spatial modulation of the eddy-current magnetic field induced by sequential defects, the center positions of subsequent defects #2 to #5 are successively located at x = 261, x = 407, x = 554, and x = 700.

**Figure 15:**
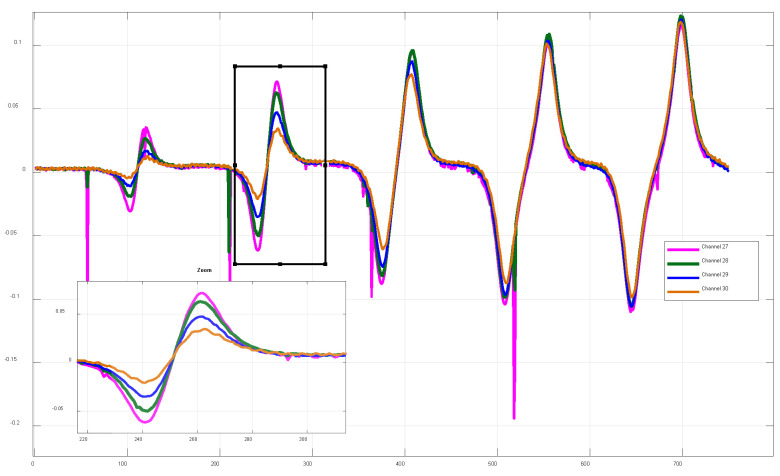
Spatial variation in defect-imaging impedance for channels 27–30.

To further elucidate the coupling mechanism between sensing elements adjacent to defects, the complex impedance data for defect imaging from channels 27 to 30 are analyzed, as shown in Figure 15. It can be observed that adjacent channels (27–30) exhibit highly consistent response patterns to the same defect, while displaying orderly variations in amplitude. All defect responses present stable and repeatable bipolar structures. Among these channels, channel 28 exhibits the optimal response in terms of peak amplitude, waveform symmetry, and stability, whereas the amplitude differences among channels reflect the lateral spatial distribution of the defect within the sensitive region of the array.

In contrast, channel 30 is spatially offset from the defect center, resulting in reduced electromagnetic field coupling efficiency with the defect region and, consequently, a significant attenuation of the measured impedance amplitude. The signal peak decreases markedly from 0.0712 V at the central region to 0.032 V. This amplitude attenuation reveals the spatial response characteristics of the sensor array and its high sensitivity to lateral variations in defect position. The M-DECA probe demonstrates good synchronization and stability in spatial sampling along the scanning direction, enabling defect-induced eddy-current perturbations to be consistently projected onto multiple adjacent channels.

## 4. Conclusions

This study addresses the challenges of weak signal responses, insufficient spatial resolvability, and difficulties in quantitative characterization of near-surface defects in metal substrates. A multiplexed differential eddy-current array probe with spatiotemporal coding features and an equilateral triangular configuration (M-DECA probe) is proposed and experimentally validated. The detection performance of the proposed probe is systematically evaluated through theoretical modeling, numerical simulations, and experimental investigations. The main conclusions can be summarized as follows: (1) a spatiotemporally coded differential eddy-current array probe structure and sampling mechanism are proposed; (2) an electromagnetic coupling model between the probe and the metal substrate is established, revealing the excitation frequency selection mechanism; (3) experimental results confirm the frequency selection predicted by simulations and demonstrate the imaging performance advantages of the proposed probe; and (4) quantitative inversion and accuracy evaluation of near-surface defect sizes are achieved.

The findings of this work provide a valuable reference for the application of differential eddy-current array testing techniques in the detection and quantitative evaluation of near-surface, low-contrast defects. Future work will extend the proposed approach to more complex defect morphologies and multi-parameter conditions to further enhance its engineering applicability and robustness.

## Figures and Tables

**Table 1:** Simulation parameters.

Parameters	Size [mm]	Conductivity [S/m]	Relative Permittivity	Parameters	Size [mm]
Air-shed	40 × 50 [mm]^2^	$\sigma_{1}$ = 1 [S/m]	$\mu_{1}=1$	$h_{2}$	0.3
Aluminum substrate	5 × 50 [mm]^2^	$\sigma_{2}$ = 3.774 × 10^7^ [S/m]	$\mu_{2}=1$	$h_{3}$	1.0
Coil Unit	coil_id: 0.8	5.998 × 10^7^ [S/m]	1	$h_{4}$	5.0
	coil_od: 2.0				
	coil_h: 1.0				
detect	0.3 × 1.0 [mm]^2^	$\sigma_{3}$ = 1 [S/m]	$\mu_{3}=1$	$d_{1}$	50.0
$h_{1}$	31.5	/	/	$d_{2}$	1.0

**Table 2:** Key parameters of the related circuit.

Module	Component	Key Timing Parameter	Typical Value
Excitation source	AD9834	Wake-up time	1 ms
Voltage-controlled amplifier	VCA810	0.1% settling time	5–8 ns
Power amplifier	ADA4870	0.1% settling time	55–82 ns
Excitation amplifier	AD829	0.1% settling time	90 ns
ADC driver	ADA4932	0.1% settling time	9 ns
Multi-channel switch	AD732	Switching time	2 ns
Single-channel processing period	-	-	1 ms
Full scanning period	-	-	64 ms

Note: The 0.1% settling time is used to characterize the transient stability of the analog front-end under high-precision amplitude extraction conditions.

**Table 3:** Comparison between inverted defect sizes and actual measurements with errors.

	Fre	10 KHz	30 Khz	50 KHz	60 Khz	80 Khz	1 Mhz	Optimal Detectable Size	Inversion Error
Defect									
#1 [d = 2]	Range	\	\	[103, 118]	[80, 98]	[68, 106]	\	2.127	6.35%
	Diameter			2.127	2.322	4.294			
#2 [d = 4]	Range	[61, 75]	[150, 182]	[239, 268]	[222, 257]	[238, 284]	\	3.8286	4.29%
	Diameter	6.286	6.176	3.8286	4.257	5.198			
#3 [d = 6]	Range	[103, 121]	[244, 293]	[375, 419]	[366, 420]	[399, 472]	\	6.2392	3.98%
	Diameter	8.082	9.457	6.2392	6.966	8.249			
#4 [d = 10]	Range	[144, 168]	[342, 404]	[498, 571]	[510, 583]	[568, 660]	\	10.3514	3.50%
	Diameter	10.776	11.966	10.3514	9.417	10.396			
#5 [d = 14]	Range	[187, 213]	[439, 509]	[626, 719]	[658, 744]	[731, 838]	\	13.1874	5.80%
	Diameter	11.674	13.51	13.1874	11.094	12.091			

Note: “\” indicates that the data are severely blurred and accurate values cannot be obtained.

## Data Availability

Dataset available on request from the authors.
